# Prediction of Calving to Conception Interval Length Using Algorithmic Analysis of Endometrial mRNA Expression in Bovine

**DOI:** 10.3390/ani11010236

**Published:** 2021-01-19

**Authors:** Dawid Tobolski, Karolina Łukasik, Agnieszka Bacławska, Dariusz Jan Skarżyński, Miel Hostens, Wojciech Barański

**Affiliations:** 1 Department of Internal Diseases with Clinic, Faculty of Veterinary Medicine, University of Warmia and Mazury, Oczapowskiego 14, 10-957 Olsztyn, Poland; 2 Department of Reproductive Immunology and Pathology, Institute of Animal Reproduction and Food Research, Polish Academy of Sciences, Tuwima 10, 10-748 Olsztyn, Poland; k.lukasik@pan.olsztyn.pl (K.Ł.); a.baclawska@pan.olsztyn.pl (A.B.); d.skarzynski@pan.olsztyn.pl (D.J.S.); 3 Department of Farm Animal Health, University of Utrecht, Yalelaan 7, 3584 CL Utrecht, The Netherlands; m.m.hostens@uu.nl; 4 Department of Animal Reproduction with Clinic, Faculty of Veterinary Medicine, University of Warmia and Mazury, Oczapowskiego 14, 10-957 Olsztyn, Poland; wojbar@uwm.edu.pl

**Keywords:** postpartum diseases, activin, inhibin, cytokines, endometrium, subclinical endometritis, cow

## Abstract

**Simple Summary:**

Our study aimed to develop the unsupervised clustering model based solely on selected markers to investigate the association between calving conception interval length, subclinical endometritis, and endometrial gene expression. An algorithmic analysis of endometrial gene expression showed a higher predictive ability to identify cows exhibiting excellent fertility than previously used methods, highlighting the correlation between *INHBA/INHA* and calving–conception interval length.

**Abstract:**

After parturition, the uterus undergoes significant reconstruction, allows the endometrium to create an environment for subsequent embryo development. Here, we used an unsupervised algorithmic approach to select characteristic endometrial mRNA expression patterns of proposed markers and investigate each marker’s role as an individual indicator of reproductive success. Clinically healthy cows at a sixth week postpartum were examined, the percentage of neutrophils (PMNs%) in the cytological smear was calculated, and an endometrial biopsy was taken for qPCR. Based on pregnancy examination, cows were divided into three groups: Pregnant before 100 days postpartum (P100, n = 11), pregnant between 100–200-day (P200, n = 14), and culled (C, n = 10). Animals were also classified based on two PMNs% thresholds > 5% PMNs and > 10% PMNs. The expression of *IL1B, IL6, CXCL8,* and *IL17A* was higher in >10%PMNs. The expression of PTGS1 was higher in the P200 compared to P100. Upregulation of inhibin A subunit (*INHA*) and downregulation of inhibin β A subunit (*INHBA*) were observed in the P100. *INHBA/INHA* ratio was the most accurate linear predictor of the calving-to-conception interval. The application of the k-means algorithm allowed the identification of five unique expression patterns. The sensitivity and specificity of predicting allocation to P100 were 81% and 79%. We also documented the low efficiency of genes associated with subclinical endometritis and PMNs% in determining reproductive capability. These results suggested the presence of distinctive expression patterns in 6 weeks postpartum, correlated with cows’ reproductive capacity. Furthermore, we proposed the *INHBA/INHA* ratio as an indicator of calving-to-conception interval length.

## 1. Introduction

The uterus undergoes involution after parturition that finishes around day 40–50 postpartum (PP). Endometrial return to the condition before pregnancy may be delayed due to uterine inflammatory processes such as metritis, clinical (CE), and subclinical (SE) endometritis or other causes related to calving ease, breed, metabolic status, and age of the cows [1]. Thus, the development of diseases in the postpartum period is considered the primary risk factor for an increased period between parturition and following conception (CCI) [2]. Minimizing the length of CCI could increase cow’s milk yield relative to parturition and feed cost, increase calves’ number, reduce culling and cost of recurrent insemination [3]. Therefore, the development of a forecasting tool for CCI’s length is highly anticipated by veterinarians and farmers.

Subclinical endometritis is described as an inflammation of the endometrium manifested by an elevated level of neutrophils in the absence of purulent vaginal discharge. Generally, SE’s effect on reproduction performance was evaluated based on the days diagnosed relative to parturition, increasing with time and neutrophils’ threshold. Subclinical endometritis diagnosed at day 20–33 PP increased median days open from 112 in healthy cows to 141, from 100 to 162 when diagnosed at day 34–47 PP [4], and from 118 to 206 when diagnosed at day 40–60 PP [5]. A recent study predicting the pregnancy status using the percentage of polymorphonuclear leukocytes (PMNs%) evaluated between 42- and 49-days PP as a diagnostic method revealed a sensitivity of 36.9, 39, and 53.6% for day 100, 150, and 200, respectively. The percentage of PMNs showed the highest sums of sensitivity and specificity along with the lowest hazard ratio and odds ratio as a predictor of pregnancy status up to 100-day PP (compared to transrectal palpation measurement of the cervical diameter, ultrasonographic measurement of the fluid in uterus score, vaginoscopic detection of external uterine orifice hyperemia, vaginal discharge score). The PMNs percent, together with other tested predictors, shows a low predictive value to detect cows able to implant embryo before 100 days PP [6].

The diagnosis of metritis or CE is straightforward due to the visible discharge from the vagina and fluid presence in the uterus lumen during an ultrasound examination [2]. However, in the absence of clinical symptoms, evaluation of endometrial conditions is difficult, time-consuming, and almost impossible at a farm site. A commonly-used method to evaluate the status of the endometrium is to count the number of neutrophils infiltrating the mucosa layer done by a cytobrush [4], low-volume uterine lavage [5], cytotape [7], or uterine secretions [8]. Nevertheless, these listed diagnostic tools allow to collect and evaluate only superficial cells and mucus. Thus, a better method to investigate ongoing molecular changes during the endometrium’s late postpartum recovery is a biopsy [9,10,11], as well as further research is needed to determine the association between biopsy findings and reproductive performance of the cows [12]. Currently used diagnostic methods focus on finding indicators describing the ongoing inflammatory process and correlations between PMNs and inflammatory mediators. Determination of the tissue markers of the cow’s likelihood to become pregnant would allow the creation of tools for monitoring both the health of the individual cow as well as herd level.

Expression of inflammatory mediators, including cytokines, chemokines, is altered in the endometrium after parturition and during SE and CE. Previous studies showed that a high number of PMNs is associated with increased expression of interleukin 1 beta (*IL1B*), Interleukin 6 (*IL6*), Interleukin 8 (*CXCL8*), and Tumor Necrosis Factor between the fifth and eighth-week PP [13,14]. Increased expression of cytokines and chemokines in this period is not associated with SE or CE at week 4 PP but with an ongoing inflammatory process. The same authors also suggested that an excessive immune response in the late puerperium may cause poor reproduction performance in dairy cows [15]. Therefore, it is crucial to investigate the association of increased interleukins expression in the late postpartum period with the probability of cows becoming pregnant.

Endometrial production of prostaglandins (PGs) plays an essential role in pregnancy, parturition, and subsequent uterine involution. Production of PGs is controlled mostly by prostaglandin-endoperoxide synthase 2 (*PTGS2*) and prostaglandin-endoperoxide synthase 1 (*PTGS1*), which provide substrates for the downstream reactions. Prostaglandin F and E synthases (*PRXL2B*, *PTGES*) are responsible for prostaglandins F (PGF) and E (PGE) synthesis. They are essential for the correct progression of the estrus cycle and return to physiological cyclicity after parturition [16]. Cows with increased PMNs in endometrial smear had lower PGF concentrations and higher PGE than healthy cows in the fourth week postpartum, and the ratio of PGE to PGF production may be one of the causes of delayed involution or endometrial restoration [17].

Another group of factors potentially associated with involution and endometrial tissue remodeling is the activin and periostin (*POSTN*) pathways [18,19]. Two Inhibin βA subunits (*INHBA*) form activin A, while the *INHBA* subunit and the inhibin α subunit (*INHA*) form inhibin A, which, together with follistatin (*FST*) are antagonists of activin A [20]. Until now, studies confirmed a regulatory role of activins and inhibins in ovarian function, folliculogenesis, and crucial role in the proper function of the female reproductive tract [21,22]. Studies on other species underlined the importance of activin A and its subunit as a monomer in the inflammatory response, wound healing, scar formation, and organ fibrosis [23,24]. There are limited data relating to the endometrial expression levels and their local function. Salilew-Wondim [18] suggested *INHBA* among 28 other genes as a potential marker of endometrial inflammation, including change of *INHBA* expression as a potential mechanism of molecular dysregulation of uterine receptivity and homeostasis [18].

Hence, we hypothesized that both subclinical endometritis as a disease and markers proposed for its diagnosis are useful predictors of future reproductive performance. In this study, we aimed to use an unsupervised algorithmic approach to select characteristic endometrial mRNA expression patterns of proposed markers in 6 weeks postpartum and investigate the role of each marker as an indicator of reproductive success.

## 2. Materials and Methods

### 2.1. Ethic Statement

The Local Ethics Committee for animal experiments in Olsztyn approved the research by resolution No. 49/2016.

### 2.2. Animals and Study Design

The study was performed on one dairy farm with Polish Holstein-Friesian cows. The cows ranged from the first to the fifth lactation with an average 305 d milk production over 9000 kg/cow. The cows were housed in a free-stall barn and fed with a partial mixed ration. Animals were milked by means of a voluntary milking system and artificially inseminated by a single AI technician after 60 days of voluntary waiting period at every visible heat until pregnancy detection or culling. Heat detection was performed three times a day for 30 min by one observer. The outcome of each insemination was noted for the estimation of bulls’ fertility. The quality of bulls’ semen did not differ and met standards for the production of frozen bovine semen. Daily milk yield for the first 60 days of lactation was obtained from the DeLaval ALPRO database (Supplementary Materials Table S1).

The first vaginoscopy was performed between 21 to 29-day PP. Cows that showed any signs of clinical endometritis (CE) or received treatment for mastitis, lameness, and pneumovagina were excluded from the study. Forty-three remaining animals were re-examined between day 35 and 42 postpartum. Based on inspection of the tail, vulva, and vaginoscopy, five cows were diagnosed as CE and treated with intrauterine antibiotics; therefore, they were excluded from the study (3/5—clear mucus containing flecks of white pus, 1/5—<50% pus in mucus, 1/5—>50% pus in mucus). Ultrasound examination of the genital tract was done to exclude cows with ovarian cysts (n = 1), estrus at the time of examination (n = 2). The diameter of the cervix and uterine horns was measured (Table S1). Finally, thirty-five cows were included in the study cohort. Blood was collected from the coccygeal vein to determine the cow’s metabolic condition at the time of sampling (Table S1). Progesterone concentration in serum was measured to assess the influence of active corpus luteum on endometrial gene expression.

### 2.3. Sample Collection

Blood samples were collected from the coccygeal vein using the VACUETTE blood collection system (VACUETTE^®^ TUBE 9 mL CAT Serum Clot Activator, Greiner Bio-One, Kremsmünster, Austria). Collected tubes were placed on ice and transported to the laboratory. Blood serum was separated by centrifugation at 4000 rpm at 4 °C for 20 min (Beckman Coulter, J-6 MC, Brea, CA, USA). The serum was transferred to 2 mL Eppendorf tubes, placed in the ultra-freezer, and stored at −80 °C until further analysis. The concentration of total cholesterol (TC), triglyceride (TG), non-esterified fatty acids (NEFA), β-Hydroxybutyrate (BHB) was measured using a biochemical analyzer (Cormay Group, ACCENT-200, Łomianki, Poland). Progesterone levels in the samples were measured using the Radio Immuno Assay method in the β-radiation counter (Pharmacia, Wallace 1410, Finland) (Table S1).

Uterine cytology samples were obtained at the second examination by the cytobrush method (Cervical Brush, Zarys International Group, Zabrze, Poland). Briefly, the brush was mounted on the mandrel and inserted into a sterile metal catheter. For the protection of the brush from vaginal contamination, the sterile gloves for rectal examination were used. The entire setup was inserted into the cow’s genital tract and passed through the cervical canal under the second hand’s control placed in the rectum. At the uterine end of the cervix, the glove protecting catheter was punctured. The catheter was carefully inserted into the uterus’ right horn, and the sample was taken by clockwise rotation of the rodded brush. After sample collection, the brush was pulled into the catheter, and the complete setup was gently drawn back from the cow’s genital tract. The brush was pushed from the catheter, and cytological material was transferred on the glass slide by rolling on it.

Uterine biopsy sample collection was obtained with biopsy forceps six weeks postpartum (Kevorkian’s uterine biopsy forceps, Hauptner Herberholz, Solingen, Germany). Sterile forceps were placed into a rectal glove and passed in the same manner as a catheter into the uterine horn. Forceps were opened, and the uterine wall was gently pressed into the jaw that was closed. Endometrial tissue samples were obtained by quick retraction of the forceps from the uterus and immediately placed into tubes for low-temperature storage. Subsequently, at the farm, tubes were dipped into liquid nitrogen and stored. Next, the samples were placed in the ultra-freezer and stored at −80 °C until further analysis. Samples weighed around 50 mg. Only one tissue fragment was taken to minimize the influence of biopsy on the reproductive performance of cows. Ultrasound pregnancy diagnosis (Honda HS-1500 Ultrasound, Toyohashi, Japan) was performed every fourth week by a single veterinarian after sample collection until 200 days postpartum or the cow was culled.

### 2.4. Cytological Examination

The smear was fixed by air dry and dyed with Romanowski type staining (Hemavet, Kolchem, Łódź, Poland). The percentage of neutrophils was calculated based on the examination of 300 cells at 400× magnification under the light microscope. One observer evaluated all samples.

### 2.5. RNA Isolation

Endometrial samples were taken from the storage in tubes kept at −80 °C and homogenized using a ceramic mortar in liquid nitrogen to a form of fine tissue powder. Subsequently, samples were moved into a 1.5 mL Eppendorf tube with 400 microliters of phenozol, stored for 30 min, vortexed for 1 min, and centrifugated. According to the producer’s protocol, total mRNA from endometrial samples was isolated with the Total RNA Mini Plus kit (A&A Biotechnology, Gdynia, Poland, #036-100). The isolated product was stored at −80 °C. Quantity and quality of the isolated total mRNA were measured with the NanoDrop spectrometer. The same amount (1000 ng) of mRNA from each sample was reverse transcribed using Maxima First Strand cDNA Synthesis Kit (Thermo Fisher, Waltham, MA, USA, #K1641). Reverse transcription was done according to the manufacturer’s protocol. cDNA was frozen at −20 °C until further analysis.

### 2.6. Real-Time PCR

For Real-time PCR analysis, ABI Prism 7900 sequence detection system (Applied Biosystems, Life Technologies, Foster City, CA, USA) was used with Maxima SYBR Green/ROX qPCR Master Mix (2X) (Thermo Scientific, Waltham, MA, USA, #K0222). The samples’ preparation was done according to the manufacturer’s protocol using 15 ng cDNA per well in a total of 10 microliters of the reaction mix. PCR was performed in duplicates for every sample using 384-well plates (MicroAmp™ Optical 384-Well Reaction Plate with Barcode, #4309849). Two reference genes (*C20RF29, SLC30A6*) were used to normalize and calculate arbitrary gene relation units [25]. The primers were designed with web-based software Primer BLAST (http://www.ncbi.nlm.nih.gov/tools/primer-blast). The size of the amplified fragments and sequences of primers are presented in Supplementary Material Table S2. Miner software (http://miner.ewindup.info) was used for the relative quantification of mRNA.

### 2.7. Statistical and Machine Learning Analysis of Reproduction Success Based on mRNA Expression

Collected data were analyzed using Python 3.7 programming language (Python Software Foundation, https://www.python.org/) and R 3.6.0 (R Core Team, 2017, https://www.r-project.org/).

For the supervised data analysis approach, two thresholds of the neutrophil percentage of 5% [26] (n = 35, <5% PMNs = 20 and >5% PMNs = 15) and 10% [4] (n = 35, <10% PMNs = 26 and >10% PMNs = 9) were selected to check the effect of subclinical endometritis (SE) on mRNA expression. The normality assumption of studied parameters was tested using the Shapiro–Wilk test, and Levene’s test was used to evaluate the equality of variances. A non-parametric Mann–Whitney U test was used to compare mRNA expression differences in the thresholds mentioned above.

Correlation between gene expression data, calving-to-conception interval (CCI), and PMNs percent in smears were calculated using the SciPy library spearman r function. Matplotlib and seaborn libraries were used to create a graphical representation of the data. Differences between groups were presented as fold difference between medians of normalized mRNA expression.

In order to check the differences in mRNA expression related to pregnancy outcomes, animals were divided: Pregnant animals up to 100 days (P100, n = 11), pregnant between 100- and 200-days PP (P200, n = 14) and other animals as culled (C, n = 10, including two cows sold to another farm at day 70 and 92 postpartum, two cows culled at 145 and 173 because of lameness and six not pregnant after 200 days).

Multivariable cox proportional hazards regression (package survival, function coxph) was used to determine the association of mRNA expression of selected genes with pregnancy risk to 200 days in milk. First, the model containing all selected genes was established, and variables with *p* < 0.2 were retained in the analysis. Next, using manual stepwise backward elimination variables with a *p* < 0.05 established model characterized by the lowest Akaike information criterion. The Schoenfeld residuals were used to check assumptions for cox proportional hazards regression (R package survival, function cox.zph).

The linear regression model (Python package SciPy) was performed using the log2 transformed mRNA expression for P100 and P200 groups predicting CCI. The minimalistic and highly accurate linear regression model was established based on the Akaike information criterion, correlations between mRNA expression, and studied genes’ biological function. A simple model including a low number of genes increases the applicability of current study in veterinary practice by reducing the analysis’s expense and duration.

Logistic regression (R function glm) was performed to predict pregnant animals up to 100 days after parturition. The backward elimination method (R function step) was used to select a model representing the lowest Akaike information criterion (R function AIC) and has the highest sensitivity and specificity. The forward elimination method was used to choose a model characterized by a small number of variables while maintaining high sensitivity and specificity.

Unsupervised data analysis was used to identify specific mRNA expression patterns of selected genes and link them to future reproductive performance. The analysis was carried out in two stages with machine learning algorithms (K-nearest Neighbor Clustering + Hierarchical Clustering and Random Forest Clustering + Hierarchical Clustering).

A K-nearest neighbor clustering (K-means, R function kmeans) algorithm was performed in 10,000 iterations on a matrix of all studied cows and log2 mRNA genes in a range of 2–10 clusters. The optimal number of clusters was determined using the elbow method and the Silhouette method. Initial clustering validation allows as to narrow k ranged to 3–6. Then, hierarchical clustering (R function hclust) was done in 1000 iterations until consensus was achieved to create dendrograms. 1-Pearson correlation distance (R function distanceMatrix) was used to calculate the distance matrix followed by the Complete-linkage clustering algorithm. Clusters were automatically divided into those that predict reproductive success in more than or equal to 50% of animals and those below 50% to determine the effectiveness of predicting cows’ pregnancy up to 100 days after parturition. This approach allowed calculating the sensitivity and specificity of the models. The Least Significant Difference (LSD) test was used to determine which genes were particularly crucial in assigning the animal to the cluster for the model with the highest sensitivity, specificity, and low akaike information criterion (AIC). The clustering results for the best-suited model were presented in the form of an mRNA expression heatmap (R package ComplexHeatmap). Belonging to the pregnancy group and the 5% and 10% SE thresholds were added after clustering analysis as descriptive data and presented next to the heatmap for each cow taking part in the experiment.

Random Forest algorithm (R function randomForest) was run in unsupervised mode and 10,000 trees to generate the proximity matrix. The optimal number of cuts was determined using the “elbow” method and the Silhouette method. The hierarchical tree was cut in the range of 3–6 branches, narrowed based on initial clustering validation. Next, the proximity matrix was converted to the distance matrix. Hierarchical clustering (R function hclust) was done in 1000 iterations until consensus was achieved using the wards method. Clusters were divided into those that predict pregnancy in the same method presented in K-means, and sensitivity and specificity were calculated. The importance of each mRNA gene expression in Random Forest was investigated by a mean decrease in the Gini index.

## 3. Results

Descriptive data including a diameter of the cervix, a diameter of left and right uterine horns, mean daily milk yield for the first 60 days of lactation, and concentrations of total cholesterol, triglycerides, non-esterified fatty acids, β-Hydroxybutyrate across all studied groups are presented in Table S1. There were no significant differences between the studied groups in the presented biochemical parameters. There was no significant difference in the fertility of used bulls measured in the whole-farm population and the study population.

### 3.1. Expression of Selected Genes

#### 3.1.1. mRNA Expression of IL1B, IL6, CXCL8, and IL17A

The mRNA expression of *IL1B*, *IL6*, *CXCL8*, and *IL17A* was significantly different between <10% PMNs and >10% PMNs groups (*p* < 0.05). *IL1B* and *IL6* expression were 2-fold higher in >10% PMNs group, and transcription of *CXCL8* and *IL17A* were 3.5-fold and 3-fold higher for >10% PMNs group.

**Figure 1:**
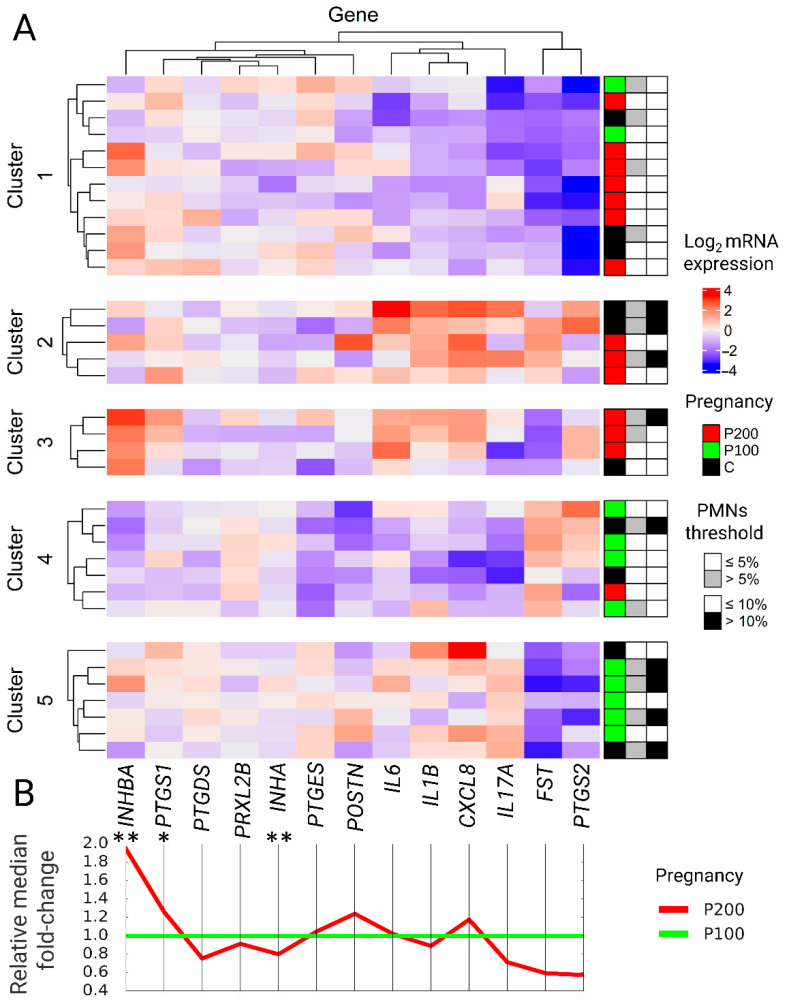
(**A**) Heat map of mRNA expression divided into 5 clusters. Cows with a similar expression pattern of marker genes were cluster together. Belonging to the pregnancy group, the 5% and 10% SE thresholds are presented next to the heatmap for each cow (row). (**B**) Median mRNA expression fold-change relative to the P100 group. * *p* < 0.05, ** *p* < 0.01.

There were no significant differences between groups using the threshold of 5% of PMNs (*p* > 0.05) as well as pregnant and not pregnant (*p* > 0.05) (Figure 1B).

#### 3.1.2. mRNA Expression of INHA, INHBA, FST, and POSTN

Comparing the expression of *INHA* and *INHBA* in the P100 and P200 groups, significant differences were observed (*p* < 0.05). Transcription of *INHA* in group P100 was 1.3-fold higher compared to group P200 (*p* < 0.05), while a 2-fold lower expression was observed in the P100 group for *INHBA* compared to P200 (*p* < 0.05). The expression of *FST* and *POSTN* did not differ between groups. *INHBA*/*INHA* ratio was 3.5-fold lower in group P100 compared to P200 (*p* < 0.05) (Figure 1B).

*INHBA*, *INHA*, *FST*, and *POSTN* expressions were similar for 5% and 10% of PMN’s thresholds (*p* > 0.05).

#### 3.1.3. mRNA Expression of PRXL2B, PTGDS, PTGES, PTGS1, and PTGS2

The expression of *PTGS1* was 1.3-fold higher in the P200 group compering to P100 (*p* < 0.05). The mRNA expression of *PRXL2B*, *PTGDS*, *PTGES*, and *PTGS2* was similar, either using pregnancy or both cytological thresholds for group comparison (*p* > 0.05) (Figure 1B).

#### 3.1.4. Correlation Analysis

The percentage of PMNs was correlated with the expression of *CXCL8*, *IL17A*, and *IL1B*. The rest of the analyzed genes were not significantly correlated with PMN’s in the sixth week postpartum. CCI was negatively correlated with *INHA* while positively correlated with *INHBA* and *PTGS1* expression. Expression of *IL1B* and *CXCL8* was positively correlated with all interleukins’ genes, while *IL6* and *IL17A* were correlated with other interleukins but not with each other (Table 1).

### 3.2. Conventional Models and Unsupervised Clustering

#### 3.2.1. Multivariable Cox Hazard Proportional Regression and Linear Regression

**Figure 2:**
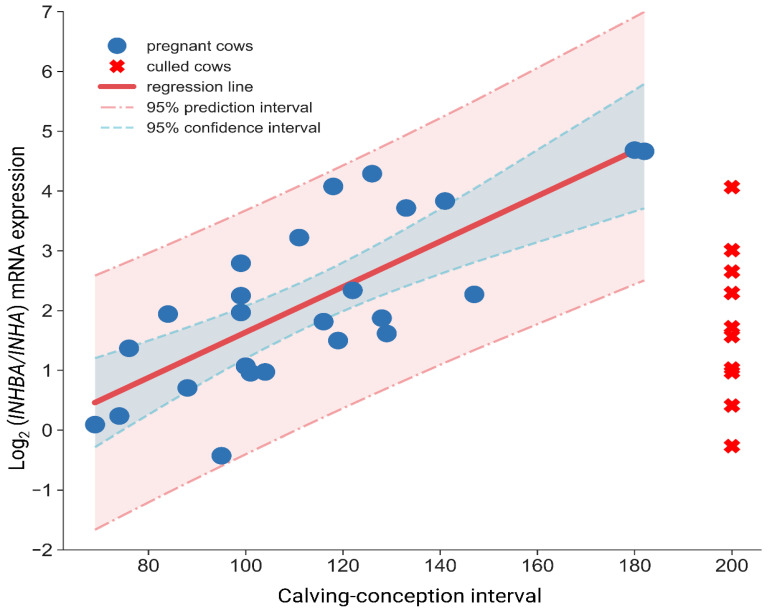
Relationship between calving conception interval and mRNA expression ratio of INHBA/INHA established using linear regression analysis.

Cox hazard proportional regression indicates that higher endometrial mRNA expression of *INHBA* and *PTGS1* decreases the risk of being pregnant given day PP, while *INHA* and *POSTN* increase the risk of being pregnant (Table 2). Using *INHBA*/*INHA* in linear regression, we could predict CCI with R^2^ = 0.575 (Figure 2, Table 3).

#### 3.2.2. Logistic Regression and Clustering

The most accurate model in predicting pregnancy up to 100 days PP used mRNA expression of *INHA*, *INHBA*, *FST*, *POSTN*, *IL1B*, *IL6*, *CXCL8*, and *PTGES* achieved sensitivity and specificity equal 100%. Whereas the model using the forward elimination method consisting of *INHA*, *INHBA*, and *POSTN* achieved a 73% sensitivity and a specificity of 87% (Table 4).

K-means and random forest analysis gave similar predicting accuracies. The optimal mRNA expression pattern clusters estimated by elbow and Silhouette methods for both algorithms was 3, while the highest sensitivity and specificity were achieved by dividing into 4 clusters for random forest and 5 clusters for K-means. An additional increase in cluster numbers over five significantly increases the Akaike information criterion (Table 4). Mean decrease in Gini index for 4 clusters random forest was *IL1B*—2.81, *CXCL8*—2.79, *PTGS2*—2.78, *INHBA*—2.70, *IL6*—2.69, *FST*—2.64, *PTGES*—2.64, *IL17A*—2.62, *POSTN*—2.58, *PRXL2B*—2.57, *PTGDS*—2.56, *PTGS1*—2.55, and *INHA*—2.55. The highest sensitivity—81%, and specificity—79%, were noted by the K-means algorithm, presented in the form of a heatmap (Figure 1A).

Cluster 1 had the lowest expression of *PTGS2*, *IL1B*, *IL6*, low expression of *CXCL8,* and *IL17A*, no cow had >10% PMNs SE (0/12), and 4/12 had >5% PMNs SE. It was also characterized by high expression of *INHBA* and *PTGES*. Nevertheless, the low PMNs% did not turn into high fertility up to 100 days PP (2 pregnant/12).

Cluster 2 and 3 were characterized by the highest *IL1B*, *IL6*, *CXCL8*, and *PTGS1*, lowest expression of *INHA*, and the small number of cows pregnant up to 100 days PP (0 pregnant/9). Cluster 2 includes animals with the highest *IL17A* and a higher proportion of cows at risk of >10% PMNs SE (3/5). Cluster 3 consisted of animals with the highest expression of *INHBA* and *PTGS1*.

Cluster 4 and 5 were characterized by the highest *INHA* expression, lowest *INHBA* and *PTGS1* expression, and highest number of cows pregnant up to 100 days PP (9 pregnant/14). Cluster 4 includes animals that had low *IL1B*, *IL6*, and lowest *IL17A*, *CXCL8* expression, and a low likelihood of >10% PMNs SE (1/7). Cluster 5 consisted of animals with high *IL1B*, *IL17A*, and *CXCL8* expression but lower IL6 expression, then cluster 2 and 3 and an elevated risk of having >10% PMNs SE (4/7).

## 4. Discussion

Expression of selected genes as an indicator of future reproduction performance and subclinical endometritis.

Markers that were evaluated in the present study were combined in three groups: (1) Interleukins, (2) enzymes responsible for prostaglandins metabolism, and (3) factors involved in tissue restoration—taking into consideration two levels of PMNs (5% and 10%) for the diagnosis of SE and prediction of future reproduction performance.

(1) It was found that all examined interleukins were significantly higher in cows with a threshold of 10%, indicating a more robust inflammatory response. The present study agreed with previous findings showing increased expression of interleukins and its significant correlation with the number of immune cells in the endometrial biopsy or the evaluated cytological smear [15,27,28]. Correlation between PMNs% and interleukins expression was also confirmed when K-means clustering data were analyzed. Cows characterized by higher interleukins expression and >10% PMNs tend to cluster together. Formerly the difference in expression of *IL1B* between healthy and SE cows was not always manifested and depended highly on the experimental design or postpartum time [12,13,26,27]. While in agreement with the previous studies, we have found that a higher PMNs% in endometrial smear during SE is correlated with increasing endometrial expression of *CXCL8*. These data prove the usefulness of *CXCL8* mRNA expression measurement as a marker of linear change in tissue infiltration by neutrophils [13,14,15,29]. Interleukin 17A also proved to be a promising marker of SE and increased PMNs%. Johnson [30] showed the increased expression of interleukin 17A in animals with SE compared to the healthy, and the same significant relationship was observed in the present study for animals with >10% PMNs. Elevated expression of Interleukin 6 in the group of >10% PMNs was also observed in the current study what agrees with previous results analyzing variations between healthy and SE cows [14,30,31]. Our goal was to select specific interleukins that could serve as potent and certain markers validating the condition of subclinical endometritis, which undoubtedly succeeded. On the other hand, we were surprised by the absence of an apparent link between subclinical endometritis at the level of interleukin expression and future reproductive performance. This discrepancy could be easily noticed by clustering where cows with low interleukin expression were grouped at the same time, showing no signs of increased fertility (cluster 1), and on the contrary, a group of cows with high interleukin expression and PMNs% could have been pregnant before 100 days PP (cluster 5). The presented data indicate that interleukins are excellent markers of the ongoing inflammatory process or PMNs%. Nevertheless, low expression of interleukins or increase during disease and subsequent decrease due to the healing could not be used as a marker of future reproduction success. Therefore, our results could justify at mRNA expression level why cytobrush PMNs% used as a predictor of pregnancy status up to 100-day PP had low diagnostic performance (sensitivity 36.9%) [6] and indicates the need to select new and more accurate forecasting methods.

(2) There is a lack of agreement in the literature about whether the expression of enzymes involved in prostaglandin metabolism could be used as a valid marker of inflammation in subclinical endometritis. *PTGS1* is rarely examined in the endometrium because its role in physiological regulations of the bovine estrous cycle and pregnancy is limited, and its expression is almost not changed [16]. Studies have not confirmed significant differences in *PTGS1* expression between healthy and cows with SE at 4, 5, and 6 weeks PP [15], which agrees with our findings. While the results obtained in our research show that animals pregnant before day 100 postpartum had lower *PTGS1* mRNA expression in the 6th-week postpartum. A change in the prostaglandin E synthesis pathway may explain the positive correlation between CCI and PTGS1 expression, initiating an imbalance in prostaglandin E to F production [17]. Under physiological status, endometrial *PTGS2* expression is positively correlated with *PTGES* expression in the endometrium during the estrous cycle and early pregnancy [16]. In the present study, the correlation was negative. The observed result might indicate that in the late stage of the uterus involution or under pathological status (i.e., subclinical endometritis), the endometrial expression pattern of *PTGS2* is different than during the physiological estrus cycle. It has been found previously that mRNA *PTGS2* is highly expressed only in the clinical form of endometritis [15]. In contrast, Ledgard [32] showed that higher *PTGS2* expression had an outcome of a greater pregnancy rate after embryo transfer. Similarly, higher expression of *PTGS2* was observed at 20-day of pregnancy comparing to 20-day of the estrus cycle in the caruncular endometrium [33]. In the present study, we found numerically higher levels of this gene expression in animals pregnant before 100-day postpartum and significantly lower expression in the first k-means cluster associated with subfertility. This variation was not correlated with the presence of SE but might contribute to infertility. A similar relationship was found in repeat breeder cows where control cows had higher *PTGS2* mRNA expression [34].

(3) Studies conducted by Salilew-Wondim [18] indicated the possibility of *POSTN* being a candidate gene that allows distinguishing healthy animals from cows with SE due to its role in tissue remodeling and fibrosis tissue formation. *POSTN* is also suggested as a potential mediator of conceptus elongation and recognition of the embryo in small ruminants by a dam [35]. *POSTN* expression remains high during epithelial inflammation or prolonged wound healing [36]. On the other hand, the level of *POSTN* is rapidly growing and subsequently falls equally quickly to a low level in quickly healing wounds or non-inflammatory tissue [37]. We considered this gene as a possible indicator of normal uterine involution. We did not find significant changes in the expression level between P100 and P200 groups, only numerically higher expression in P200 animals. Using logistic regression to predict animals pregnant before day 100 postpartum and linear regression predicting CCI *POSTN* showed a significant contribution to models. However, the highest-efficiency models’ main components, both linear and binomial, in predicting future reproductive performance were INHBA and INHA expression. So far, there has been no research on the possibility of using *INHA* and *INHBA* as an indicator of normal or delayed uterine involution. Being aware that calving can damage uterine tissue and specific bacteria enter uterine lumen at that time. We hypothesized that the uterus during involution might use similar molecular mechanisms to wound healing. The role of activin in tissue remodeling during wound healing is extensively studied. Inhibition of activin action by over-expression of follistatin causes a delay in wound repair while reducing scar tissue formation [38]. Therefore, higher expression of *INHBA* may indicate the ongoing active remodeling of the tissue. Microarrays revealed *INHBA* as one of the most profoundly altered genes in the endometrial inflammation caused by lipopolysaccharides (LPS) [18]. The present study showed that animals ready to get pregnant before day 100 PP had lower endometrial mRNA expression of *INHBA* than the subfertile group, which may signal the termination of histological involution and return of endometrium to the status before pregnancy. Increased expression of *INHA* occurring in animals with higher reproduction performance can cause competition for the *INHBA* subunit. Thus, the switching mechanism between these two expression patterns may limit the formation of activin A in favor of Inhibin A working as a “molecular clock” of endometrial restoration. Highlighting the gap in knowledge and possible limitations of the study. More research is needed to determine the exact function of analyzed genes, the level of their protein products, the role of inhibin A and activin A in the endometrium after delivery and throughout the whole involution period, as well as validation of the findings on a large number of animals and farms. Using the proposed hypothesis at the mRNA expression level, we suggest that the *INHBA*/*INHA* ratio (Figure 2) is a far better indicator of the present status of the endometrium than the use of *INHBA* and *INHA* separately. Obtained results show that this parameter can be applied as an indicator of endometrial restoration, and we found a linear relationship between *INHBA*/*INHA* ratio and future reproductive performance, namely lower ratio characterized cows exhibiting excellent fertility and shorter CCI.

Prediction of gene expression patterns associated with future reproduction performance using Machine Learning Algorithms.

In this study, the logistic regression model (*INHA, INHBA, FST, POSTN, IL1B, IL6, CXCL8,* and *PTGES*) identified by the backward elimination method had sensitivity and specificity equal to 100%. This highly accurate prediction is burdened with high bias. Conventional statistics, especially binomial logistic regression, has many limitations when analyzing correlated data. When applied to the new dataset, models created in that way frequently show far lower predictive abilities. The selection of machine learning algorithms has been proposed to interpret correlated data [39]. Until now, models take into consideration qualitative features in the form of mixed [40] or binomial models, bringing additional effort and cost of data collection. Our model is based solely on gene expression. The decision to use unsupervised algorithms was directed to find characteristic mRNA gene expression patterns of selected markers occurring six weeks after delivery and identify which of them are associated with improved and reduced fertility. K-means and random forest clustering approaches had similar sensitivity and specificity with the superiority of the first one. K-means method in the present study resulted in 5 distinct gene expression groups in sixth-week PP. The unsupervised division of cows confirmed the usefulness of endometrial gene expression as a prospective reproductive indicator. Two clusters were associated with high reproduction performance, a 3 cluster with reduced fertility. Extended validation of our result can offer a new way to assess information about the endometrial condition and the ability for embryo reception.

## 5. Conclusions

We provided evidence that lower expression of *INHBA* and higher INHA expression in the sixth week postpartum characterized cows showing better reproduction performance, and usefulness of *INHBA*/*INHA* ratio to predict CCI length. The k-means algorithm application allowed identifying five unique expression patterns of the studied genes at 6th-week postpartum, of which two were characterized by high reproduction performance. We also documented the low efficiency of gene markers associated with SE and PMNs% in determining reproductive capability.

## Figures and Tables

**Table 1:** Spearman rank correlation (upper triangle) and *p*-values (lower triangle) for the mRNA expression of studied markers.

	*IL1B*	*IL6*	*CXCL8*	*IL17A*	*INHA*	*INHBA*	*FST*	*POSTN*	*PRXL2B*	*PTGDS*	*PTGES*	*PTGS1*	*PTGS2*	PMNs%	CCI
***IL1B***	*IL1B*	0.500	0.834	0.590									0.504	0.380	
***IL6***	0.002	*IL6*	0.437			0.383							0.457		
***CXCL8***	0.001	0.009	*CXCL8*	0.484									0.374	0.522	
***IL17A***	0.001		0.003	*IL17A*										0.430	
***INHA***					*INHA*						0.356				−0.491
***INHBA***		0.023				*INHBA*	−0.449	0.510							0.654
***FST***						0.007	*FST*		0.400		−0.396		0.434		
***POSTN***						0.002		*POSTN*							
***PRXL2B***							0.017		*PRXL2B*	−0.395					
***PTGDS***									0.019	*PTGDS*					
***PTGES***					0.036		0.019				*PTGES*		−0.417		
***PTGS1***												*PTGS1*			0.480
***PTGS2***	0.002	0.006	0.027				0.009				0.013		*PTGS2*		
**PMNs%**	0.024		0.001	0.010										PMNs%	
**CCI**					0.013	0.001						0.015			CCI

**Table 2:** Results of the multivariable Cox hazard proportional model in the studied population (n = 35) showing the effects of mRNA gene expression on the time to pregnancy to 200 DIM (backward elimination method).

Model	β	HR	SE	95% CI		*p*-Value
				0.025	0.975	
*INHA*	1.2528	3.5001	0.5778	1.1280	10.8609	0.030
*INHBA*	−1.1534	0.3156	0.321	0.1676	0.5941	<0.001
*POSTN*	0.7849	2.1923	0.2880	0.4561	1.2467	0.006
*PTGS1*	−0.7929	0.4525	0.4039	0.2051	0.9988	0.049

Concordance = 0.778, Likelihood ratio test = 26.76, Wald test = 17.45, Score (logrank) test = 21.33, Akaike information criterion = 97.25, Bayesian information criterion = 102.13.

**Table 3:** Linear regression model for calving conception interval prediction (INHBA/INHA ratio).

Model	Stand. β	Unstand. β	SE.	95% CI		*p*-Value
				0.025	0.975	
Intercept		80.9842	6.9968	66.5102	95.4582	<0.001
*INHBA/INHA*	0.7583	15.1439	2.7147	9.5281	20.7598	<0.001

R: 0.76, R^2^: 0.57, Adjusted R^2^: 0.56, RMSE: 19.22, Model *p*-value < 0.001.

**Table 4:** Comparison of sensitivity, specificity, and precision of logistic regression, K-means clustering (k = 3–6), and random forest clustering (k = 3–6) in predicting allocation to P100.

Model	AIC	BIC	Sensitivity	Specificity	Precision
Logistic regression					
*INHA*	37.46	40.57	0.45	0.87	0.63
*INHA + INHBA*	35.00	39.67	0.55	0.87	0.66
*INHA + INHBA + POSTN*	31.94	38.17	0.73	0.87	0.73
*INHA+ INHBA+ FST+ POSTN+ IL1B+ IL6+ CXCL8+ PTGES*	18.00	31.99	1	1	1
K-Means Clustering					
K3	360.17	420.17	0.36	0.87	0.57
K4	356.89	437.77	0.36	0.87	0.57
K5	360.70	461.80	0.81	0.79	0.64
K6	365.19	486.51	0.81	0.79	0.64
Random Forest Clustering					
K3	380.27	440.93	0.27	0.87	0.5
K4	363.66	444.54	0.82	0.71	0.66
K5	364.87	465.97	0.82	0.75	0.53
K6	374.94	496.26	0.73	0.79	0.73

AIC: Akaike information criterion, BIC: Bayesian information criterion, AUC: Area under the curve.

## Supplementary Materials

The following are available online at https://www.mdpi.com/2076-2615/11/1/236/s1, Table S1: The diameter of the cervix and diameter of left and right uterine horns, mean daily milk yield for the first 60 days of lactation and blood concentrations of total cholesterol, triglycerides, non-esterified fatty acids, β-Hydroxybutyrate across all studied groups (Mean ± SD); Table S2: Selected genes, primer forward, and reverse sequences used for RT-qPCR.
