# Supplementary material for: Prediction of Calving to Conception Interval Length Using Algorithmic Analysis of Endometrial mRNA Expression in Bovine
Source: Animals (Basel). 2021 Jan 19;11(1):236. doi: 10.3390/ani11010236 (PMC7835770; doi:10.3390/ani11010236)
Supplement: Supplementary file 1 [file animals-11-00236-s001.pdf]

Table S1. The diameter of the cervix and diameter of left and right uterine horns, mean daily milk yield for the first 60 days of lactation and blood concentrations of total cholesterol, triglycerides, non-esterified fatty acids, β-Hydroxybutyrate across all studied groups (Mean±SD). P100- pregnant animals up to 100 days postpartum, P200- pregnant between 100 and 200 days postpartum, C- culled (including two cows sold to other farms at day 70 and 92 postpartum, two cows culled at 145 and 173 because of lameness and six not pregnant after 200 days), ⌀C- cervix diameter, ⌀LH- left horn diameter, ⌀RH- right horn diameter, MY60- mean daily milk yield for first 60 days of lactation, TC- total cholesterol, TG- triglyceride, NEFA- non-esterified fatty acids, BHBA- β-Hydroxybutyrate, P^4-progesterone.

**Parameter**		**P100**	**P200**	**C**	**<5% PMNs**	**>5% PMNs**	**<10% PMNs**	**>10% PMNs**
**n**		**11**	**14**	**10**	**20**	**15**	**26**	**9**
⌀C	mm	32.18 ± 3.12	34.50 ± 3.67	32.80 ± 3.74	33.90 ± 4.14	32.47 ± 2.56	33.85 ± 3.76	31.67 ± 2.50
⌀LH	mm	28.45 ± 3.36	29.07 ± 4.71	27.90 ± 3.73	29.50 ± 4.17	27.27 ± 3.39	29.19 ± 4.08	26.67 ± 3.08
⌀RH	mm	27.55 ± 3.62	29.29 ± 4.68	28.40 ± 4.19	29.85 ± 4.18	26.67 ± 3.52	29.11 ± 4.02	26.67 ± 4.30
MY60	kg	37.36 ± 6.36	38.79 ± 6.36	34.30 ± 9.65	36.75 ± 7.63	37.47 ± 7.70	36.77 ± 7.17	37.89 ± 8.98
TC	mmol/L	4.02 ± 0.93	4.43 ± 0.75	3.66 ± 1.10	4.13 ± 0.97	4.02 ± 0.96	4.11 ± 0.89	4.02 ± 1.16
TG	mmol/L	0.18 ± 0.031	0.19 ± 0.022	0.18 ± 0.015	0.19 ± 0.025	0.18 ± 0.019	0.19 ± 0.024	0.17 ± 0.019
NEFA	mmol/L	0.63 ± 0.39	0.55 ± 0.25	0.54 ± 0.14	0.59 ± 0.33	0.54 ± 0.16	0.59 ± 0.30	0.52 ± 0.17
BHB	mmol/L	1.03 ± 0.33	0.97 ± 0.29	0.99 ± 0.17	0.98 ± 0.26	1.01 ± 0.29	0.97 ± 0.24	1.07 ± 0.35
P^4	ng/mL	0.87 ± 1.14	0.71 ± 1.05	0.54 ± 0.10	0.46 ± 0.69	0.95 ± 1.21	0.57 ± 0.85	0.91 ± 1.23

Table S2. Selected genes, primer forward, and reverse sequences used for RT-qPCR.

Gene	NCBI GenBank Accession number	Primer forward sequence<br>Primer reverse sequence	Fragment size
IL1B	NM_174093.1	AAGGCTCTCCACCTCCTCTC<br>TTTGGGGTCTACTTCCTCCA	186
IL6	NM_173923.2	TCTGGGTTCAATCAGGCGAT<br>TGTTTGTGGCTGGAGTGGTT	196
CXCL8	NM_173925.2	GTTGCTCTCTTGGCAGCTTT<br>GGTGGAAAGGTGTGGAATGT	118
IL17A	NM_001008412.1	GCTCTTGTGAAGGCAGGAGT<br>ATTGCGGTGGAGAGTCCAAG	177
PTGDS	NM_174791.4	ACACTTCACCACCTTTGCCA<br>GCACTTGTCAGTCTTCGGCA	76
PTGS1	NM_001105323	CAACTGCACCATCCCTGAGA<br>GGGGATAAGGTTGGAACGCA	175
PTGS2	NM_174445.2	AATCTTCCAGTCGCAGTAG<br>TTGAGGCAGTGTTGATGAT	376
PTGES	NM_174443.2	GAAGAAGGCTTTTGCCAACCC<br>AAGACCAGGAAGTGCATCCG	198
PRXL2B	NM_001040598.1	GCCAAAGGTGGTGATAAAGTGC<br>GAGCATGCCTCTTCATCGCA	146
INHBA	NM_174363.2	GGACGGAGGGCAGAAATGAA<br>TTCCTGGCTGTGCCTGATTC	80
INHA	NM_174094.4	CGGCTGCAGGTGCCA<br>CTGGGATGGCTGGAACACAT	86
FST	NM_175801.3	CTATGCTAGCGAGTGTGCCA<br>GTGTCTTCCGAAATGGAGTTGC	99
POSTN	NM_001040479.1	CCCCATAACTGTCTACAAGCCA<br>TGACCTTGGTGACCTCTTCTTGC	200
C20RF29	XM_582695.5	CCTTCAAGAGCCCCCTGT<br>GGGTCCTTTTCCAACTCTCC	64
SLC30A6	NM_001075766.1	TGATGAGGAAACCTAGCCCTGCC<br>TCGGGCTGCTCCAAAAAGCGT	142
